# A systematic review of the pharmacokinetics of classical serotonergic psychedelic compounds in healthy adult subjects

**DOI:** 10.1177/02698811261453938

**Published:** 2026-05-29

**Authors:** Elliot Hampsey, Kirsty Martin, Michail Kalfas, Louis Benson, Sirid Wihlborg, Luke Jelen, Allan H. Young, James Rucker

**Affiliations:** 1 Institute of Psychiatry, Psychology & Neuroscience (IoPPN), Kings College London, UK; 2 South London and Maudsley NHS Foundation Trust, UK; 3 Department of Brain Sciences, Imperial College London, UK

**Keywords:** pharmacokinetics, classical psychedelics, systematic review

## Abstract

**Introduction:**

Despite renewed investigations into classical psychedelic compounds, their pharmacokinetic profiles remain incompletely understood.

**Methods:**

This systematic review collated data from healthy adult volunteers on the pharmacokinetic properties of lysergic acid diethylamide (LSD), psilocybin, dimethyltryptamine (DMT), mescaline, and 5-methoxy-*N,N*-dimethyltryptamine (5-MEO-DMT).

**Results:**

We identified 32 eligible trials. LSD was the most studied compound, followed by DMT, split between intravenous (IV) and oral formulations. Psilocybin was also frequently studied. Mescaline was reported in two trials, with IV LSD, IV psilocybin, inhalation 5-MEO-DMT, and intranasal 5-MEO-DMT reported in single studies. Key findings include dose proportional *C*_max_ values for LSD and psilocybin, alongside differences between oral and IV formulations of DMT that may be clinically significant.

**Discussion:**

This systematic review highlights key variations in absorption, distribution, and elimination between the studied compounds that may have important implications in both clinical and research settings.

## Introduction

Classical psychedelics are a class of psychoactive compounds that act as broad agonists at serotonin receptors (with the exception of 5-HT3) in the brain (Halberstadt, 2015). Aside from lysergic acid diethylamide (LSD), which is semi-synthetic, the main classical psychedelics all occur naturally and have been used for spiritual, medicinal, and recreational purposes by geographically separated cultures since antiquity, highlighting an intimate and long-standing interest in their effects (George et al., 2022). Western medical use dates back to the late 19th century, when Silas Weir Mitchell and colleagues documented their experiences with mescaline, and further still when one considers Humphry Davy’s fin-de-siécle experiments with nitrous oxide in the 18th century. Albert Hofmann’s serendipitous discovery of LSD in 1943 drove the first wave of clinical research, prematurely terminated after 1970 due to an emerging wave of political and cultural opprobrium towards psychoactive drugs in general (Weleff et al., 2023). After a 30-year pause, reinvigoration of clinical and mechanistic interest has gathered pace since the turn of the millennium (Kishon et al., 2024).

The core molecular structure of different psychedelic drugs follows a common pattern: they have an aromatic ring system connected by a two-carbon chain to a basic amine group. The specific aromatic group present distinguishes the two major groups of psychedelics: tryptamines and phenethylamines (Kwan et al., 2022).

The major tryptamine psychedelics are psilocybin, LSD, and *N,N*-dimethyltryptamine (*N,N*-DMT). Psilocybin and DMT are, structurally, simple tryptamines, whilst LSD is a more complex form of tryptamine, classified as an ergolide. Psilocybin is not orally active, but is rapidly dephosphorylated in the liver to psilocin, which is. DMT is broken down by visceral monoamine oxidase when given orally. Thus, it is given intravenously or in conjunction with inhibitors of visceral monoamine oxidase such as the harmaline preparations that are a component of traditional shamanistic preparations like ayahuasca (Carbonaro and Gatch, 2016). 5-methoxy-*N,N*-dimethyltryptamine (5-MeO-DMT) is closely related to DMT (Reckweg et al., 2021).

The major phenethylamine psychedelic is mescaline (3,4,5-trimethoxyphenethylamine), which is found in two cacti species indigenous to Central and North America. The subjective experience is similar to other classical psychedelics. Synthetic derivatives of mescaline, for example, 4-bromo-2,5-dimethoxyphenethylamine (2C-B), are numerous and varied, many developed by the U.S. chemist Alexander Shulgin, and described in detail in his book *PIHKAL* (Shulgin and Shulgin, 2010). Due to the number of psychoactive phenethylamines (and tryptamines) that Shulgin synthesised and described, we do not consider them further here. Many remain completely unresearched beyond his initial self-experiment.

Protonated amine groups of serotonergic ligands typically form a salt bridge with Asp155 (D3.32) in the 5-HT2A receptor binding pocket, an interaction that anchors the ligand and contributes to agonist-induced conformational changes that enable Gq-mediated signalling and other functionally selective downstream pathways (Cummins et al., 2025). It is proposed that efficacy of an individual drug at activating the Gq system, and the downstream consequences of this on phase and timing of neuronal firing, principally distinguishes a drug that does, or does not, elicit the characteristic psychedelic experience (Wallach et al., 2023). Beyond the acute disturbance in neuronal electrical activity, the disturbance in Gq is related to downstream cascades of signalling that converge on the molecular target of rapamycin and brain-derived neurotrophic factor, resulting in longer-term changes in neuronal structure and functioning.

The subjective experience under psychedelics is characterised by an intensification and broadening of various experiential domains: perception, cognition, and emotion. At higher doses, users frequently report ‘mystical’ states and a blurring of conceptual boundaries, described more broadly as a state of ‘ego-dissolution’, where the ‘sense of self’ gives way to a broad sense of unity, interconnectedness and spiritual awareness. The range of experience also includes (more rarely) intensely negative states of mind characterised by dysphoria, anxiety, panic, paranoia, and frank psychosis.

Classical psychedelics, particularly psilocybin, LSD, and DMT, are now being used again in clinical trials for a broad range of neuropsychiatric syndromes, ranging from alcoholism and tobacco dependence through to treatment-resistant depression (TRD) and Post Traumatic Stress Disorder (PTSD). This is not dissimilar to efforts in the 1950s and 1960s, except that in the modern era psychedelics are treading a regulated, internationally recognised pathway through phase 1–3 clinical trials. Psilocybin for TRD is now in late phase 3 trials and will be considered for medical licensing by the Food and Drug Administration in the United States in late 2026. LSD, DMT, and 5-MeO-DMT for various disorders will likely follow in the years hence.

Within this context, a synthesis of the pharmacokinetic parameters of classical psychedelics is timely. A recent book chapter provides a broad narrative overview of the clinical pharmacology of classic psychedelics and 3,4-methylenedioxymethamphetamine (MDMA), covering mechanisms, pharmacokinetics, pharmacodynamics, metabolism, safety, dosing, and drug interactions (Vogt and Liechti, 2025). A previous review focussed on a limited number of articles and pharmacokinetic parameters (Holze et al., 2024). Smaller, compound-specific reviews have summarised individual compounds (Otto et al., 2025; Swieczkowski et al., 2025), but no systematic review article has, to our knowledge, synthesised all of the above. Therefore, we systematically reviewed the pharmacokinetic profiles of classical serotonergic psychedelic compounds in healthy adult participants.

## Results

### Literature search

**Figure 1.**
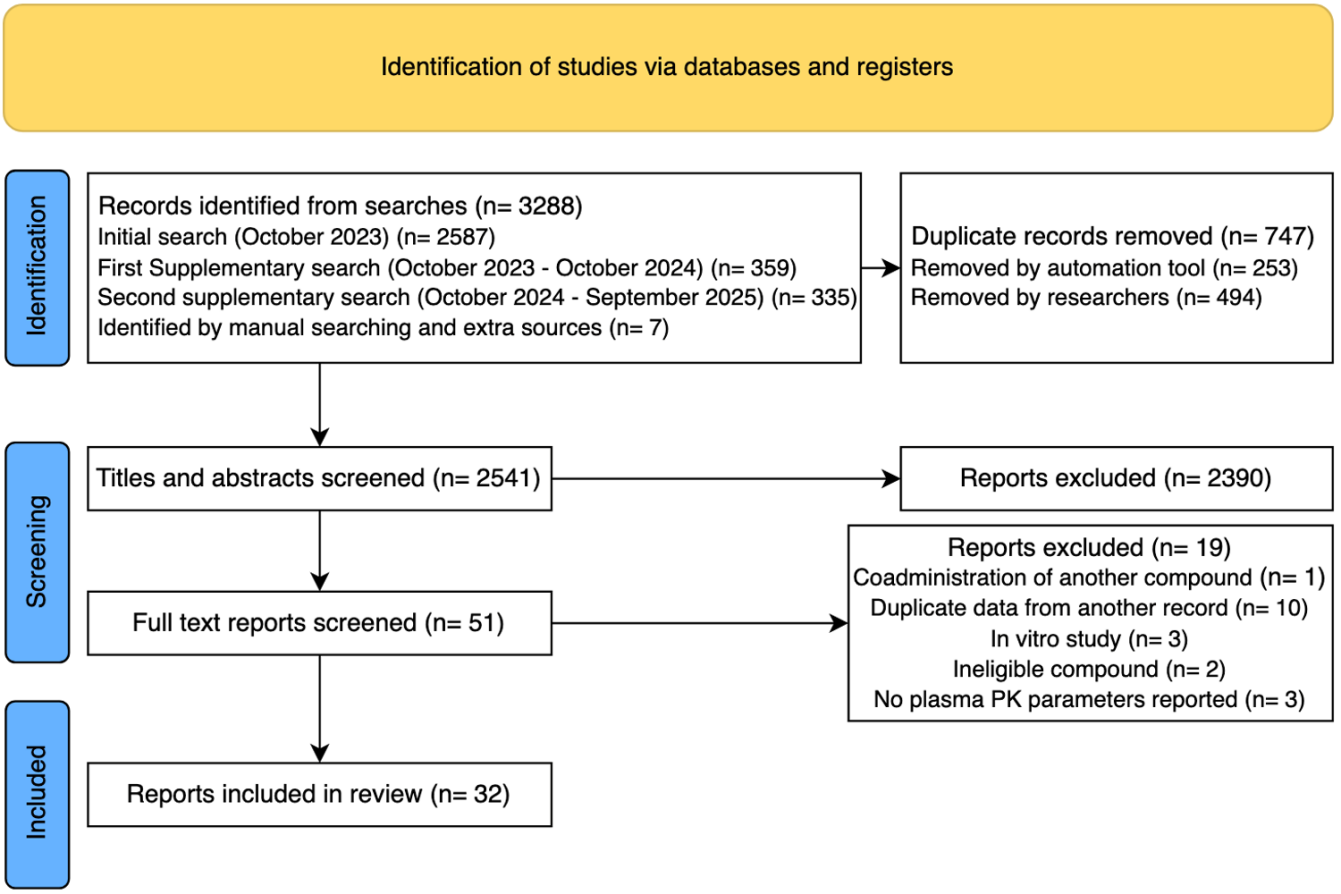
PRISMA flowchart. PRISMA flowchart detailing the systematic review process. PRISMA: Preferred Reporting Items for Systematic Reviews and Meta-Analysis.

We followed established Preferred Reporting Items for Systematic Reviews and Meta-Analysis (PRISMA) methodology for this review (Figure 1). Search results (*n* = 3288) were imported to the Rayyan platform. Records that were 100% identical after text normalisation based on title, abstract text, author, and year were removed automatically (*n* = 253). Rayyan identified additional potential duplicates based on ⩾90% similarity between title, abstract text, and year, which were decided manually (*n* = 494) by one reviewer (EH), leaving 2541 for screening.

Screening was completed in two stages by pairs of independent, blinded raters (EH, KM, and MK). Ineligible records were removed based on topic, study design, population, and the presence of obvious exclusion criteria (*n* = 2390). The full text of potentially eligible articles (*n* = 51) were screened according to our inclusion and exclusion criteria. A final list of 32 articles remained after exclusions (*n* = 19 excluded). A list of included articles, including details on study design and cohorts, is presented in Supplemental Material 2 (Arikci et al., 2025; Becker et al., 2022, 2023, 2025; Brown et al., 2017; Callaway et al., 1999; Dolder et al., 2015; Egger et al., 2025; Erne et al., 2025; Family et al., 2020, 2022; Good et al., 2023; Hasler et al., 1997; Holze et al., 2019, 2021, 2022a, 2022b, 2023; Klaiber et al., 2024; Lanaro et al., 2021; Ley et al., 2023; Lindenblatt et al., 1998; Mallaroni et al., 2023; Mason et al., 2020; Morse et al., 2025; Reckweg et al., 2021; Riba et al., 2003; Rucker et al., 2024; Strassman et al., 1996; Van Der Heijden et al., 2025; Viktorin et al., 2022; Vogt et al., 2023).

**Figure 2.**
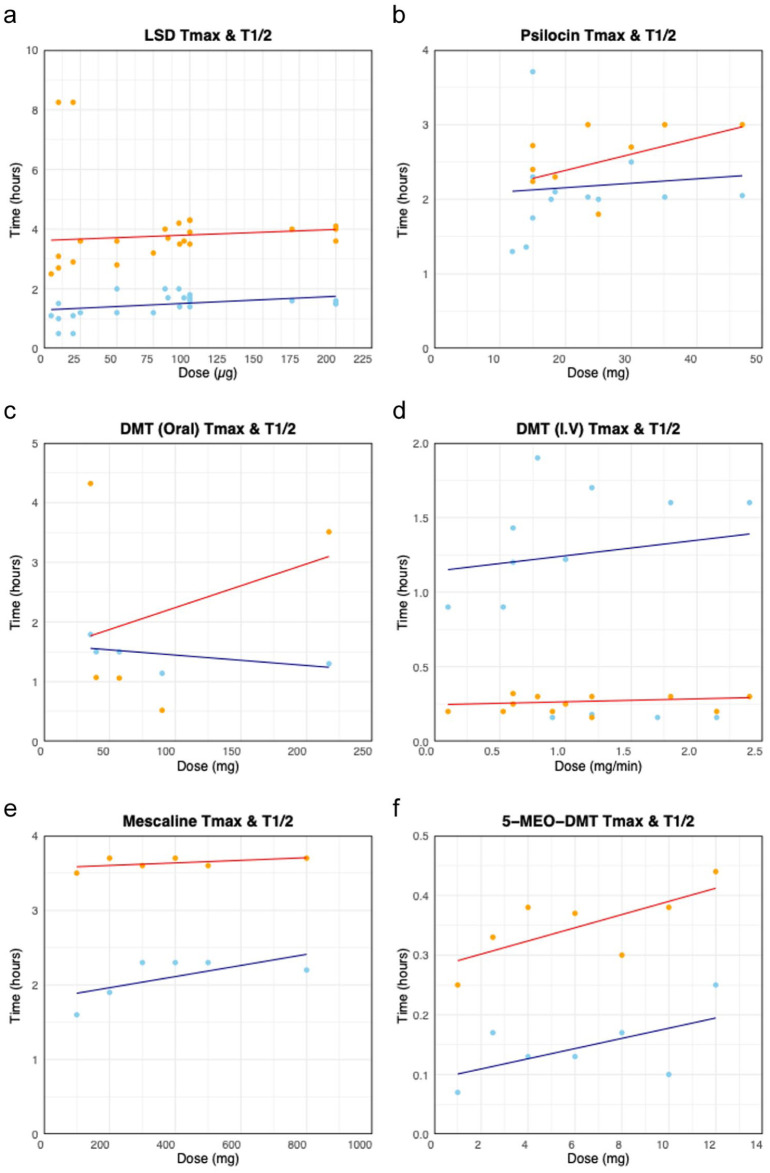
*T*_max_ and *T*_1/2_ pharmacokinetic results by compound. Scatter plots show observed trial arm mean values for *T*_max_ (blue circles, navy line) and half-life (orange circles, red line) as a function of dose for LSD (a) Psilocin (b), DMT (Oral) (c), DMT (I.V) (d), Mescaline (e), and 5-MEO-DMT (f). Lines represent simple linear regression fits weighted by trial arm sample size (*n*). Axes are compound-specific, with *x*-axis doses expressed in mg, µg, or mg/minute as appropriate.

**Figure 3.**
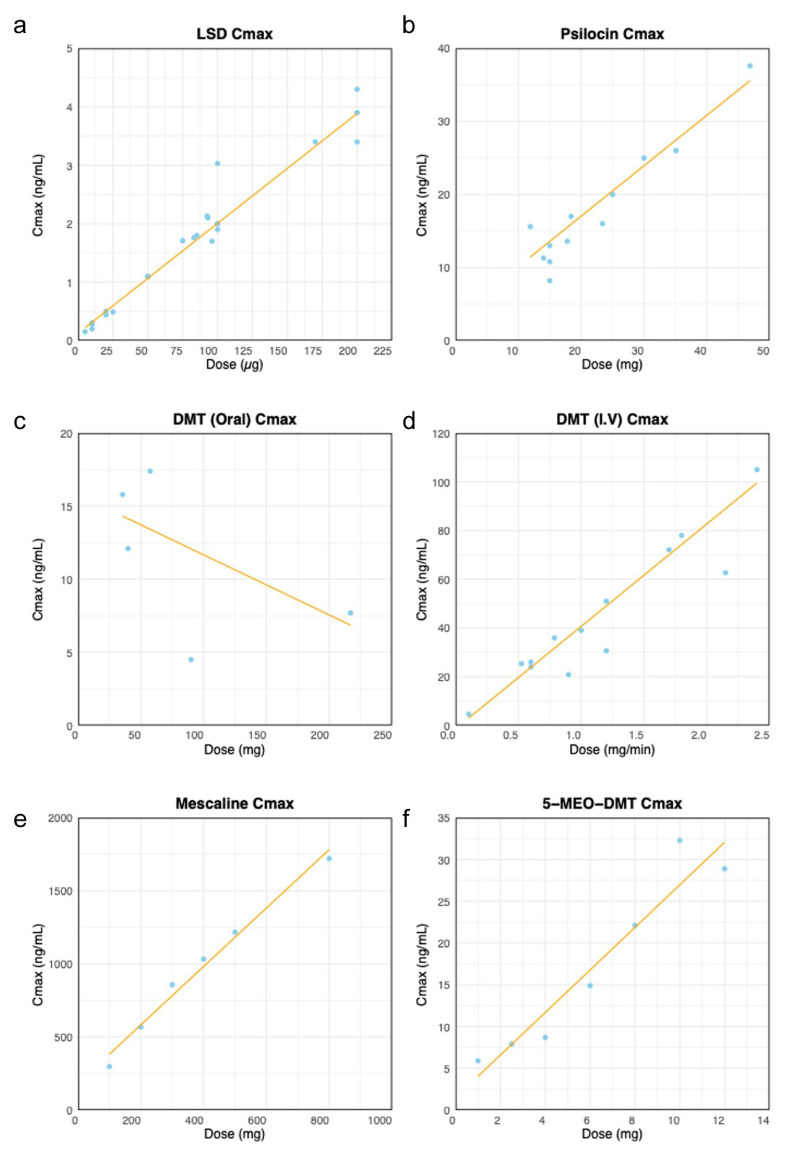
*C*_max_ pharmacokinetic results by compound. Scatter plots show observed trial arm mean values for *C*_max_ (light blue circles, orange regression line) by dose for LSD (a) Psilocin (b), DMT (Oral) (c), DMT (I.V) (d), Mescaline (e), and 5-MEO-DMT (f). Lines represent simple linear regression fits weighted by trial arm sample size (*n*). Each subplot uses a dose axis scaled to the available data for that compound. Units for *C*_max_ are ng/mL.

**Figure 4.**
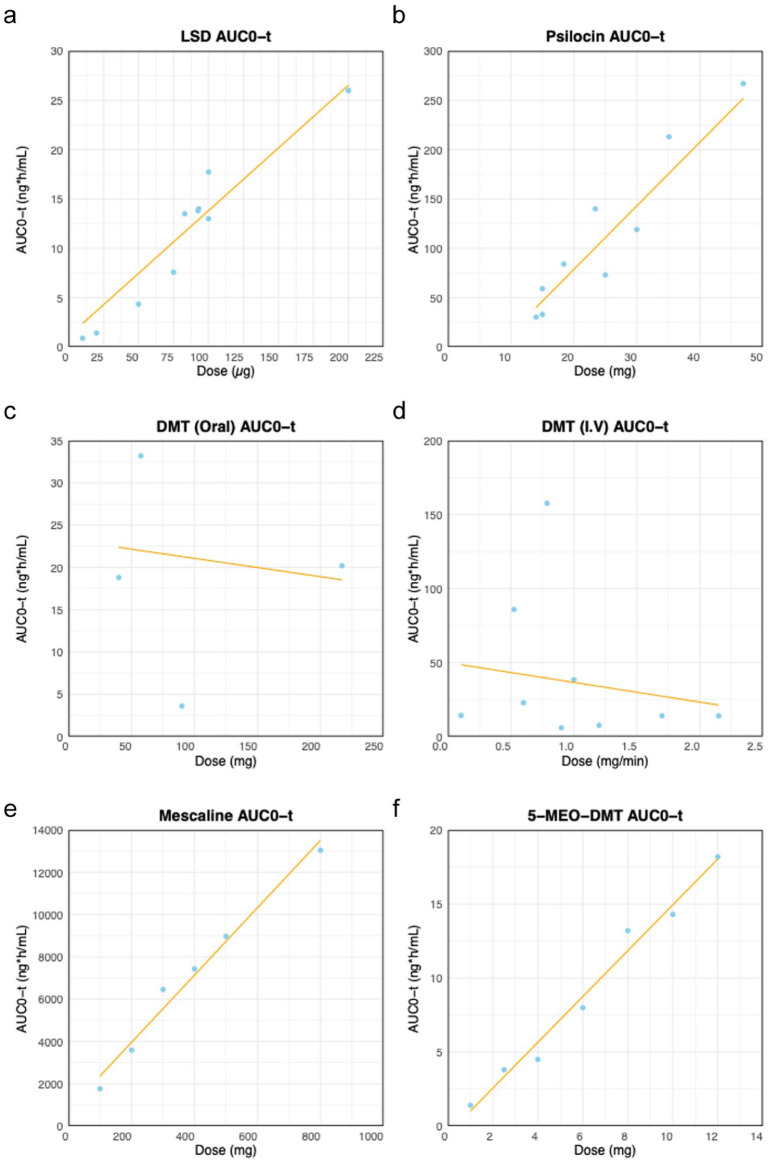
AUC_0-*t*_ pharmacokinetic results by compound. Scatter plots show observed trial arm mean values for AUC^0^-*t* (light blue circles, orange regression line) by dose for LSD (a) Psilocin (b), DMT (Oral) (c), DMT (I.V) (d), Mescaline (e), and 5-MEO-DMT (f). Lines represent simple linear regression fits weighted by trial arm sample size (*n*). Each subplot uses a dose axis scaled to the available data for that compound.

A summary of the extracted pharmacokinetic data for oral LSD, oral psilocin, IV DMT, oral DMT, inhaled and intranasal 5-MEO-DMT, and oral mescaline is presented in Supplemental Material 3, with further tables and figures available in Supplemental Material 4 to 10, respectively. Figures 2 to 4 overview key pharmacokinetic data per compound.

### Lysergic acid diethylamide

LSD was administered orally 431 times across 23 trial arms. Sample sizes ranged from 3 to 39 (mean: 18.7). Participant sex was slightly skewed, with 59.5% of analysed participants being male. Mean age ranged from 23 to 63.2 across studies (mean: 33.2). Only 12 arms reported body weight (mean: 70.92 kg). Session duration, that is, the time from administration to last observation, ranged from 6 to 24 hours (mean: 18.8).

The pharmacokinetic parameter data for oral LSD is overviewed in Supplemental Material 4. Doses ranged from 0.005 to 0.200 mg (mean: 0.082). *C*_max_ values ranged from 0.151 to 4.30 ng/mL (mean: 1.73). *T*_max_ values ranged from 0.5 to 2.0 hours (mean: 1.43). Half-life measurements ranged from 2.5 to 8.25 hours (mean: 3.98). For AUC, 0-*t* ranged from 0.891 to 26.0 ng × hour/mL (mean: 12.57), whilst 0-infinity ranged from 0.80 to 31.0 ng × hour/mL (mean: 13.26). Clearance ranged from 4.74 to 7.78 L/hour (mean: 6.81). Volume distribution ranged from 23 to 42 L (mean: 35.71).

### Psilocybin (psilocin exposure)

As psilocybin is rapidly, and near completely, converted to psilocin after oral ingestion (Passie et al., 2002), we refer to ‘psilocybin’ when referring to the compound participants are dosed with, and ‘psilocin’ for what is measured as a pharmacokinetic outcome. Psilocybin was administered orally 220 times across 12 trial arms. Sample sizes ranged from 6 to 32 (mean: 18.3). Again, participant sex skewed male (65.0%). Mean participant age ranged from 22.7 to 43.0 (mean: 34.2). Mean body weight was reported in six trial arms, with a range of 65.16 to 78.10 kg (mean: 73.23 kg). Session duration ranged from 6 to 24 hours (mean: 15.3).

Pharmacokinetic results for psilocin are summarised in Supplemental Material 5. Psilocybin doses ranged from 11.9 to 46.9 mg (mean: 22.3). *C*_max_ ranged considerably from 8.2 to 37.6 ng/mL (mean: 17.8). *T*_max_ values ranged from 1.3 to 3.7 hours (mean: 2.1). Half-life measurements had a narrow range of 1.8 to 3 hours (mean: 2.6). For AUC, 0-*t* ranged from 30.2 to 267 ng × hour/mL (mean: 113), whilst 0-infinity ranged from 32.7 to 131 ng × hour/mL (mean: 78.5). Clearance ranged from 155 to 263 L/hour (mean: 195). Volume distribution ranged from 298 to 1016 L (mean: 557).

### DMT: Intravenous

DMT was administered intravenously in 254 instances across 16 trial arms, with sample sizes ranging from 5 to 27 (mean: 15.9). Participant sex was slightly skewed, with 55.4% of analysed participants being male. Mean age ranged from 23.4 to 43.0 across studies (mean: 32.5). Seven trial arms reported body weight (mean: 72.39 kg). Session duration ranged from 2 to 10 hours (mean: 4.4).

Intravenous (IV) dosing protocols may be delivered as continuous infusions, a bolus dose, or a combination (see Supplemental Material 6). Regarding trial arms that involved continuous infusion, data are summarised in Table 1.

**Table 1.** Intravenous DMT summary.

Trial arm reference	Infusion rate (mg/min)	Dose (mg)	*N* analysed	Mean weight (kg)	*C*_max_ (ng/mL)	*T*_max_ (hours) (median)	*T*_1/2_ (hours)	AUC_0-inf_ (ng × h/mL)
Van Der Heijden et al. (2025)	0.105	39.3	8	71.6	4.60	{0.90}	0.20	13.5
Van Der Heijden et al. (2025)	0.525	197	8	70.5	25.3	{0.90}	0.20	97.8
Vogt et al. (2023)	0.600	54.0	27	NR	24	1.20	0.25	22.9
Erne et al. (2025)	0.600	72.0	22	NR	26.0	1.43	0.32	35.9
Van Der Heijden et al. (2025)	0.787	289	7	70.7	35.9	{1.90}	0.30	158
Good et al. (2023)	0.900	9.00	5	79.5	20.8	{0.16}	0.20	5.87
Vogt et al. (2023)	1.00	90.0	27	NR	39.0	1.22	0.25	38.4
Good et al. (2023)	1.20	12.0	6	59.6	30.6	{0.18}	0.16	7.58
Erne et al. (2025)	1.20	144	22	NR	51.0	1.70	0.30	67.6
Good et al. (2023)	1.70	17.0	5	74.2	72.1	{0.16}	NR	NR
Erne et al. (2025)	1.80	216	22	NR	78.0	1.60	0.30	106
Good et al. (2023)	2.15	21.5	6	80.6	62.7	{0.16}	0.20	14.0
Erne et al. (2025)	2.40	288	22	NR	105	1.60	0.30	152

DMT: dimethyltryptamine.

### DMT: Oral

DMT was administered orally 63 times across 5 trial arms, with sample sizes ranging from 8 to 15 (mean: 12.6). Participant sex was skewed, with 77.4% of analysed participants being male. Mean age ranged from 25.7 to 39.5 across studies (mean: 32.4). Only three trial arms reported body weight (mean: 69.05 kg). Session duration ranged from 8 to 24 hours (mean: 14.6).

The pharmacokinetic parameter data for oral DMT is overviewed in Supplemental Material 7. Doses ranged from 35.5 to 217 mg (mean: 87.9). *C*_max_ values ranged from 4.50 to 17.4 ng/mL (mean: 11.5). *T*_max_ values ranged from 1.14 to 1.79 hours (mean: 1.41). Half-life measurements ranged from 0.52 to 4.32 hours (mean: 2.1). For AUC, 0-*t* ranged from 3.60 to 33.2 ng × hour/mL (mean: 19.0), whilst 0-infinity ranged from 5.00 to 38.3 ng × hour/mL (mean: 21.6). Clearance ranged widely from 987 to 6720 L/hour (mean: 2950). Volume distribution ranged from 2506 to 3510 L (mean: 3122).

### Mescaline

Only two eligible studies reported data on mescaline (Klaiber et al., 2024; Ley et al., 2023). Mescaline was administered orally 96 across 6 trial arms, all with 16 participants. Participant sex was balanced at 50%. Mean age was 31.7 years. Mean body weight was not reported. Session duration ranged from 24 to 30 hours.

The pharmacokinetic parameter results for mescaline are presented in Supplemental Material 8. Mescaline doses ranged from 100 to 800 mg (mean: 383). *C*_max_ ranged considerably from 298 to 1721 ng/mL (mean: 949 ng/mL). *T*_max_ values ranged from 1.6 to 2.3 hours (mean: 2.1). Half-life measurements had a narrow range of 3.5 to 3.7 hours. For AUC, 0-*t* ranged from 1767 to 13,047 ng × hour/mL (mean: 6878), whilst 0-infinity ranged from 1805 to 13,144 ng × hour/mL (mean: 6959). Clearance ranged from 37 to 61 L/hour (mean: 51). Volume distribution ranged from 188 to 328 L (mean: 264).

### 5-Methoxy-*N,N*-dimethyltryptamine

Two trials investigated 5-MEO-DMT, one via inhalation (Reckweg et al., 2021) and one via intranasal spray (Rucker et al., 2024). The former included 22 participants in a 4-arm escalating dose trial, and the latter 31 participants in a 7-arm, escalating dose trial.

The pharmacokinetic parameter results for 5-MEO-DMT are presented in Supplemental Material 9. Regarding the Rucker et al. (2024) study, doses ranged from 1 to 12 mg (mean: 6.2). *C*_max_ ranged considerably from 5.90 to 32.3 ng/mL (mean: 17.2 ng/mL). *T*_max_ values ranged from 0.07 to 0.25 hours (mean: 0.15). Half-life measurements had a narrow range of 0.25 to 0.44 hours (mean: 0.35). For AUC, 0-*t* ranged from 1.40 to 18.2 ng × hour/mL (mean: 9.06), whilst 0-infinity ranged from 1.9 to 23.7 ng × hour/mL (mean: 11.6). Clearance ranged from 522 to 953 L/hour (mean: 658). Volume distribution was not reported. The inhalation formulation trial by Reckweg et al. (2021) did not report pharmacokinetic measurements apart from *C*_max_, which was reported to range from 0.20 to 0.97 ng/mL (mean: 0.47).

### Other formulations

Both LSD and psilocybin were also studied in IV formulations (Supplemental Material 10) in one trial arm each (Arikci et al., 2025; Hasler et al., 1997), albeit the former as LSD tartrate. IV LSD tartrate (0.080 mg slow infusion) achieved median *C*_max_ of 5.94 ng/mL at 0.16 hours, half-life of 3.8 hours, and clearance of 4.7 L/hour. IV psilocybin (1.00 mg bolus) produced a psilocin *C*_max_ of 12.9 ng/mL at 0.03 hours, half-life of 1.24 hours, and clearance of 187.6 L/hour. Sample sizes were small, with single trial arm representation for each compound.

## Discussion

### Lysergic acid diethylamide

Data for LSD suggested consistent absorption patterns, though substantial interindividual variability was observed across most parameters. Interindividual variability in LSD exposure may partly reflect pharmacogenetic differences in metabolic capacity, as CYP2D6 poor metaboliser status has been linked to increased exposure and prolonged effects, with dose reductions of approximately 50% proposed (Vizeli et al., 2021). Although not assessed here, such findings provide a plausible explanation for the variability observed. More broadly, pharmacogenetic studies suggest that variability in metabolic and transporter pathways may contribute to differential responses to other psychedelics, including psilocybin, DMT, 5-MeO-DMT, and mescaline, though existing evidence is largely preclinical and the clinical relevance of these mechanisms remains uncertain (Halman et al., 2025).

Peak plasma concentrations varied widely. The elimination half-life is broadly inline with the trajectory of subjective effects. Clearance and distribution values indicated movement beyond the plasma compartment into tissues. Study populations showed a slight gender imbalance (approximately 60% male) and a wide age variation. Doses used ranged from small subperceptual doses to high doses expected to elicit substantial subjective effects. The high variability across all pharmacokinetic parameters highlights the need for further research into genetic, physiological, and demographic factors influencing LSD metabolism.

### Psilocybin/psilocin (oral)

Data for psilocybin (psilocin) confirms more rapid elimination compared to LSD, with absorption and clearance patterns suggesting extensive tissue distribution and faster metabolism. Considerable interindividual variability was observed in peak plasma concentrations and other parameters, despite a narrower dose range than that seen with LSD.

### DMT (intravenous)

Data for IV DMT suggests distinct characteristics for this drug and route of administration. Peak plasma concentrations increased proportionally with infusion rate. The relatively short elimination half-life of IV DMT distinguishes it from longer-acting psychedelics like LSD. This may allow a variable dosing protocol responsive to individual participant’s reports of intensity or management of adverse effects. Session durations, thus, were substantially shorter than those required for LSD and psilocybin, but obviously dependent on the nature of the infusion protocol.

### DMT (oral)

Data for oral DMT differed substantially to IV DMT. DMT is metabolised by visceral monoamine oxidase, and thus oral bioavailability is dependent on coadministration with a visceral monoamine oxidase inhibitor. Depending on the efficiency of such inhibition, oral bioavailability of DMT is likely to vary. Coadministration of DMT with a monoamine oxidase inhibitor frequently induces emesis. For all these reasons, it may be particularly difficult to accurately estimate the pK parameters associated with oral DMT, and this was reflected in our findings. Absorption occurred on a timescale similar to psilocin. Half-life estimates were highly variable, yet significantly shorter than both LSD and psilocin. Session durations were substantially longer than IV protocols. The high interindividual variability across all parameters and the need to coadminister oral DMT with another drug to enable absorption suggests that oral DMT may be harder to predict and manage.

### Mescaline (oral)

Peak plasma concentrations of mescaline showed considerable interindividual variability. On average, acute mescaline effects reached maximal intensity more slowly (≈4 hours) than LSD (≈2.3 hours) and psilocin (≈2.1 hours) at equivalent doses, likely reflecting slower absorption kinetics (Ley et al., 2023). The half-life was consistent across studies, comparable to LSD but longer than psilocin and much longer than IV DMT. Clearance and distribution values indicated intermediate tissue distribution relative to other psychedelics. Session duration was nearly twice that of LSD. The very limited research base, consisting of only two small studies, restricts generalisability and underscores mescaline’s status as the least-studied classical psychedelic in contemporary clinical research despite its historical importance.

### 5-MEO-DMT (inhaled/intranasal)

Intranasal 5-MeO-DMT exhibited a rapid onset and time to peak plasma concentration (as expected), and rapid elimination. Inhaled 5-MeO-DMT appeared to have a similar time to *C*_max_; however, lack of additional reported pK data from the single study found here prevents any further comparison. 5-MeO-DMT has been conceptualised as a short-acting psychedelic. The short time to peak plasma concentration may elicit experiences that are challenging to psychologically integrate, requiring additional support around the dosing session. Currently, insufficient data exist to comment further, but larger trials of 5-MeO-DMT in patients are planned.

### Psilocybin and LSD tartrate (intravenous)

The IV administration eliminates absorption delay and first-pass metabolism, resulting in rapid onset of effects and time to peak plasma concentrations. IV formulations of psilocybin and LSD remain experimental with no obvious clinical advantage over conventional oral administration; however, they remain of use in experimental research.

## Conclusion

The aim of this systematic review was to provide an up-to-date and comprehensive synthesis of the pharmacokinetic data on the main classical serotonergic psychedelic compounds in most frequent use today. Our analysis synthesises data across these compounds, allowing for pharmacokinetic comparisons that allow the reader to consider if (and if so, then how) they may have clinical applicability as we move towards an era of licensing decisions.

## Online methods

The project was pre-registered on PROSPERO (CRD42023451781), with the reporting adhering to the PRISMA statement (Page et al., 2021). Search results were managed using the Rayyan platform, with extracted data handled in Microsoft Excel. Data visualisations were created in Python (van Rossum and Python Development Team, 2023), using the matplotlib (Hunter, 2007), Seaborn (Waskom, 2021), NumPy (Harris et al., 2020), and Pandas (The Pandas Development Team, 2024) subpackages.

### Literature search strategy

This review searched Embase, Medline, Global Health, and APA PsycINFO databases. Additionally, Google Scholar and the reference lists of included studies were also searched as additional sources of potentially eligible articles. The full-search strategy is available in Supplemental Material 1. In brief, search terms targeted human studies administering any classical psychedelic compound that reported any pharmacokinetic parameters. The full results are comprised of an initial search covering up to 1 October 2023 followed by identical supplementary searches to cover from 1 October 2023 to 1 September 2025. There were no restrictions on publication region, language, or format.

### Eligibility criteria

Inclusion criteria for eligible reports were broad, including journal articles of prospective trials reporting at least one blood pharmacokinetic parameter in healthy adult (>18) participants administered only one classical psychedelic substance in at least one session. ‘Classical psychedelic substance’, in our context, included: LSD, *N,N*-DMT, 5-MEO-DMT, psilocybin, mescaline.

Exclusion criteria included trials coadministering multiple compounds; trials including participants with psychiatric conditions, medical illnesses, substance misuse; reports not available in English; and trials that reported on urinary excretion or saliva measurements only (i.e., no blood measurements).

### Study selection and data handling

Data were extracted by pairs of independent reviewers (KM, EH, MK, SW, and LB) for all eligible records using a pre-defined extraction proforma. In brief, data were extracted in two tranches. Firstly, key details including authors, region, and compound. Secondly, we then extracted details of the analysis, cohort, and pharmacokinetic parameters. Discrepancies between extracted data between authors were resolved by a third rater.

We extracted the following pharmacokinetic parameters, standardised where appropriate to the following units: maximum concentration in ng/mL (*C*_max_); time in hours after dosing to reach *C*_max_ (*T*_max_); half-life measured in hours (*T*_1/2_); clearance in litres per hour (Cl); volume distribution in litres (*V_d_*). We also recorded area under the concentration–time curve from time zero to the last measurable concentration measured (AUC_0-*t*_); and area under the concentration–time curve from time zero to infinity (AUC_0-inf_) in ng × hour/mL.

Missing data were not imputed. Dose information reported as mg/kg was converted to mg using an assumed average weight of 70 kg. Similarly, area under the curve, volume distribution, and clearance data provided by weight, volume, and time were converted using simple arithmetic to standardised units. For pharmacokinetic parameters, variance measurements were collected in the following order of preference: standard deviation, 95% confidence interval, and range.

We used Python’s data analysis and visualisation libraries matplotlib, Seaborn, NumPy, and Pandas. Extracted *C*_max_, *T*_max_, and half-life information were first formatted into comma-separated-value (CSV) datasets. Code compiled in the Google Colab environment was used to produce figures. Confidence intervals (95%) were generated via bootstrapping with 1000 iterations to assess the reliability of the dose-proportional relationship. The resulting regression lines and confidence bands were overlaid on scatterplots of the empirical data for both compounds.

## Supplemental Material

sj-docx-1-jop-10.1177_02698811261453938 – Supplemental material for A systematic review of the pharmacokinetics of classical serotonergic psychedelic compounds in healthy adult subjects Supplemental material, sj-docx-1-jop-10.1177_02698811261453938 for A systematic review of the pharmacokinetics of classical serotonergic psychedelic compounds in healthy adult subjects by Elliot Hampsey, Kirsty Martin, Michail Kalfas, Louis Benson, Sirid Wihlborg, Luke Jelen, Allan H. Young and James Rucker in Journal of Psychopharmacology
