# Supplementary material for: A systematic review of the pharmacokinetics of classical serotonergic psychedelic compounds in healthy adult subjects
Source: J Psychopharmacol. 2026 May 29;40(8):1213–23. doi: 10.1177/02698811261453938 (PMC13415903; doi:10.1177/02698811261453938)
Supplement: sj-docx-1-jop-10.1177_02698811261453938 – Supplemental material for A systematic review of the pharmacokinetics of classical serotonergic psychedelic compounds in healthy adult subjects [file sj-docx-1-jop-10.1177_02698811261453938.docx]

**Supplementary Material 1 -**Terms for database search

("classical psychedelics" OR LSD OR psilocybin OR mescaline OR DMT OR "Lysergic acid diethylamide" OR "Dimethyltryptamine" OR "5-MEO-DMT") AND ("pharmacokinetics of" OR "pharmacokinetic profile of" OR "pharmacokinetic parameters of" OR "absorption of" OR "distribution of" OR "metabolism of" OR "elimination of" OR "bioavailability of" OR "half-life of" OR "clearance of")

**Supplementary Material 2 -** Summary Table of Trial Descriptive Statistics & Trial Details

| **Study Reference** | **Compound (Route)** | **Dose (mg) \| Infusion rate** | **N Analysed** | **Male  (%)** | **Mean Age (Years)** | **Mean Weight  (kg)** | **PK Session (Hours)** | **Compartment Model** |
| --- | --- | --- | --- | --- | --- | --- | --- | --- |
| Rucker et al 2024 | 5-MEO-DMT (Intranasal) | 1.00 | 4 | 100 | 34.0 | NR | 12 | NR |
|  |  | 2.50 | 4 | 25.0 | 33.0 |  |  |  |
|  |  | 4.00 | 4 | 75.0 | 30.0 |  |  |  |
|  |  | 6.00 | 4 | 100 | 38.0 |  |  |  |
|  |  | 8.00 | 5 | 80.0 | 40.0 |  |  |  |
|  |  | 10.0 | 5 | 80.0 | 39.0 |  |  |  |
|  |  | 12.0 | 5 | 40.0 | 31.0 |  |  |  |
| Erne et al 2025 | DMT (Intravenous) | 72.0 \| 0.6/min | 22 | 50.0 | 30.0 | NR | 3 | NCA |
|  |  | 144 \| 1.2/min |  |  |  |  |  |  |
|  |  | 216 \| 1.8/min |  |  |  |  |  |  |
|  |  | 288 \| 2.4/min |  |  |  |  |  |  |
| Good et al 2023 | DMT (Intravenous) | 9.00 \| 0.9/min | 5 | 83.3 | 34.3 | 79.5 | 4 | NCA |
|  |  | 12.0 \| 1.2/min | 6 | 33.3 | 34.5 | 59.6 |  |  |
|  |  | 17.0 \| 1.7/min | 5 | 83.3 | 43.0 | 74.2 |  |  |
|  |  | 21.5 \| 2.15/min | 6 | 83.3 | 40.0 | 80.6 |  |  |
| Strassman et al 1996 | DMT (Intravenous) | 21.0 [30s bolus] | 13 | 69.2 | 35.5 | NR | 2 | NR |
| van der Heijden et al 2025 | DMT (Intravenous) | 39.3 \| 0.105/min | 8 | 62.5 | 32.0 | 71.6 | 10 | NCA |
|  |  | 197 \| 0.525/min | 8 | 50.0 | 26.0 | 70.5 |  |  |
|  |  | 289 \| 0.787/min | 7 | 42.9 | 23.4 | 70.7 |  |  |
| Vogt et al 2023 | DMT (Intravenous) | 54.0 \| 0.600mg/min | 27 | 44.5 | 33.0 | NR | 2.5 | NCA |
|  |  | 90.0 \| 1.00mg/min |  |  |  |  |  |  |
|  |  | 15mg [45s bolus] + 54.0 \| 0.600mg/min |  |  |  |  |  |  |
|  |  | 25mg [45s bolus] + 90.0 \| 1.00mg/min |  |  |  |  |  |  |
| Callaway et al 1999 | DMT (Oral) | 35.5 | 12 | 100 | 35.9 | 74.2 | 24 | NR |
| Egger et al 2025 | DMT (Oral) | 90.0 | 8 | 56.3 | 35.0 | NR | 9 | NCA |
| Lanaro et al 2021 | DMT (Oral) | 217 | 14 | 64.3 | 39.5 | NR | 24 | NCA |
| Riba et al 2003 | DMT (Oral) | 39.8 | 14 | 83.3 | 25.7 | 66.5 | 8 | NCA |
|  |  | 57.4 | 15 |  |  |  |  |  |
| Arikci et al 2025 | LSD (Oral) | 0.083 | 20 | 50.0 | 37.0 | 70.0 | 24 | NCA |
| Becker et al 2023 | LSD (Oral) | 0.093 | 24 | 50.0 | 34.0 | 71.0 | 12 | NCA |
| Becker et al 2025 | LSD (Oral) | 0.100 | 23 | 52.2 | 31.0 | 68.0 | 24 | NCA |
| Dolder et al 2015 | LSD (Oral) | 0.200 | 16 | 50.0 | 28.6 | NR | 24 | NCA |
| Family et al 2020 | LSD (Oral) | 0.010 | 7 | 50.0 | 63.2 | NR | 12 | NCA |
|  |  | 0.020 | 8 | 75.0 | 61.6 |  |  |  |
| Family et al 2022 | LSD (Oral) | 0.050 | 3 | 100 | 28.3 | NR | 24 | NCA |
|  |  | 0.075 | 7 | 85.7 | 28.3 |  |  |  |
|  |  | 0.100 | 3 | 100 | 32.0 |  |  |  |
| Holze et al 2019 | LSD (Oral) | 0.096 | 27 | 48.1 | 28.0 | 71.0 | 12 | CA |

| **Study Reference** | **Compound (Route)** | **Dose (mg) \| Infusion rate** | **N Analysed** | **Male  (%)** | **Mean Age (Years)** | **Mean Weight  (kg)** | **PK Session (Hours)** | **Compartment Model** |
| --- | --- | --- | --- | --- | --- | --- | --- | --- |
| Holze et al 2021-A | LSD (Oral) | 0.005 | 13 | 52.2 | 23 | 70 | 6 | Mixed |
|  |  | 0.010 | 18 |  |  |  |  |  |
|  |  | 0.020 | 15 |  |  |  |  |  |
| Holze et al 2021-B | LSD (Oral) | 0.025 | 16 | 50.0 | 29.0 | 69.0 | 24 | CA |
|  |  | 0.050 |  |  |  |  |  |  |
|  |  | 0.100 |  |  |  |  |  |  |
|  |  | 0.200 |  |  |  |  |  |  |
| Holze et al 2022 | LSD (Oral) | 0.100 | 28 | 50.0 | 35.0 | NR | 24 | NCA |
|  |  | 0.200 |  |  |  |  |  |  |
| Holze et al 2023 | LSD (Oral) | 0.085 | 28 | 50.0 | 35.0 | NR | 24 | CA |
|  |  | 0.170 |  |  |  |  |  |  |
| Ley et al 2023 | LSD (Oral) | 0.093 | 32 | 50.0 | 29.0 | NR | 24 | NCA |
| Morse et al 2025 | LSD (Oral) | 0.010 | 39 | 100 | 37.4 | 85.1 | 6 | CA |
| Arikci et al 2025 | LSD Tartrate (Intravenous) | 0.080 \| 0.009/min | 20 | 50.0 | 37.0 | 70.0 | 24 | NCA |
| Klaiber et al 2024 | Mescaline (Oral) | 100 | 16 | 50.0 | 33.0 | NR | 30 | NCA |
|  |  | 200 |  |  |  |  |  |  |
|  |  | 400 |  |  |  |  |  |  |
|  |  | 800 |  |  |  |  |  |  |
| Ley et al 2023 | Mescaline (Oral) | 300 | 16 | 50.0 | 29.0 | NR | 24 | NCA |
|  |  | 500 |  |  |  |  |  |  |
| Hasler et al 1997 | Psilocybin/Psilocin (Intravenous) | 1.00 [30s bolus] | 6 | 100 | 33.0 | 74.1 | 2 | NCA |
| Becker et al 2022 | Psilocybin/Psilocin (Oral) | 25.0 | 23 | 52.2 | 34.0 | 70.0 | 7 | NCA |
| Brown et al 2017 | Psilocybin/Psilocin (Oral) | 23.4 | 12 | 83.0 | 43.0 | 78.1 | 24 | NCA |
|  |  | 35.1 | 11 |  |  |  |  |  |
|  |  | 46.9 | 10 |  |  |  |  |  |
| Hasler et al 1997 | Psilocybin/Psilocin (Oral) | 1.84 | 6 | 88.9 | 30.0 | 65.2 | 7 | NCA |
| Holze et al 2022 | Psilocybin/Psilocin (Oral) | 0.015 | 28 | 50.0 | 35.0 | NR | 24 | NCA |
|  |  | 0.030 |  |  |  |  |  |  |
| Ley et al 2023 | Psilocybin/Psilocin (Oral) | 18.4 | 32 | 50.0 | 29.0 | NR | 24 | NCA |
| Lindenblatt 1998 | Psilocybin/Psilocin (Oral) | 14.0 | 7 | NR | NR | NR | 7 | CA |
| Mallaroni et al 2023 | Psilocybin/Psilocin (Oral) | 15.0 | 21 | 50.0 | 25.0 | 69.9 | 7 | NCA |
| Mason et al 2020 | Psilocybin/Psilocin (Oral) | 11.9 | 30 | 60.0 | 22.7 | NR | 6 | NR |
| Viktorin et al 2022 | Psilocybin/Psilocin (Oral) | 17.8 | 12 | NR | 36.0 | NR | 6 | NR |

CA – Compartmental Analysis; NCA – Non-compartmental analysis

**Supplementary Material 3 –** Summary Table of Descriptive Statistics and Pharmacokinetic Parameters

| **Study Reference** | **Compound (Route)** | **Dose  (mg)** | **N Analysed** | **Male %** | **Mean Age (Years)** | **Mean Weight (Kg)** | **PK Session (Hours)** | **Cmax (ng/mL)** | **Tmax (Hours)** | **AUC 0-t (ng*h/mL)** | **AUC 0-inf (ng*h/mL)** | **T 1/2  (Hours)** |
| --- | --- | --- | --- | --- | --- | --- | --- | --- | --- | --- | --- | --- |
| Rucker et al 2024 | 5-MEO-DMT (Intranasal) | 1.00 | 4 | 100 | 34 | NR | 12 | 5.9 ±1.30 | {0.07}  (0.07-0.17) | 1.4 ±0.40 | 1.9 ±0.30 | 0.25 ±0.13 |
|  |  | 2.50 | 4 | 25.00 | 33 |  | 12 | 7.9 ±3.40 | {0.17}  (0.10-0.27) | 3.8 ±1.90 | 4.40 ±2.10 | 0.33 ±0.10 |
|  |  | 4.00 | 4 | 75.00 | 30 |  | 12 | 8.7 ±4.70 | {0.13}  (0.05-0.17) | 4.5 ±2.20 | 5 ±2.20 | 0.38 ±0.13 |
|  |  | 6.00 | 4 | 100 | 38 |  | 12 | 14.9 ±5.10 | {0.13}  (0.07-0.27) | 8 ±2.50 | 9.8 ±1.30 | 0.37 ±0.05 |
|  |  | 8.00 | 5 | 80.00 | 40 |  | 12 | 22.1 ±5.50 | {0.17} (0.10-0.32) | 13.2 ±3.70 | 14 ±4.20 | 0.30 ±0.07 |
|  |  | 10.0 | 5 | 80.00 | 39 |  | 12 | 32.3 ±15.0 | {0.10} (0.03-0.27) | 14.3 ±4.60 | 15.4 ±4.40 | 0.38 ±0.13 |
|  |  | 12.0 | 5 | 40.00 | 31 |  | 12 | 28.9 ±7.20 | {0.25} (0.17-0.50) | 18.2 ±4.00 | 23.7 ±4.7 | 0.44 ±0.15 |
| Erne et al 2025 | DMT (Intravenous) | 72.0\| 120mins \|0.6/min | 22 | 50.00 | 30 | NR | 3 | 26 [22.0-31.0] | 1.43 [1.23-1.65] | NR | 35.88* [29.45-43.73]* | 2.35 [2.32-2.40] |
|  |  | 144\| 120mins \|1.2/min |  |  |  |  |  | 51 [42.0-61.0] | 1.7 [1.50-1.93] |  | 67.58* [55.37-82.50]* | 2.37 [2.33-2.42] |
|  |  | 216\| 120mins \|1.8/min |  |  |  |  |  | 78 [63.0-96.0] | 1.6 [1.42-1.80] |  | 105.7* [84.05-133.0]* | 2.37 [2.32-2.40] |
|  |  | 288\| 120mins \|2.4/min |  |  |  |  |  | 105 [85.0-128] | 1.6 [1.35-1.90] |  | 151.63* [123.2-186.7]* | 2.38 [2.35-2.42] |
| Good et al 2023 | DMT (Intravenous) | 9.00\| 10mins \|0.9/min | 5 | 83.33 | 34.3 | 79.5 | 4 | 20.8 ±12.9 | {0.16} (0.11-0.18) | 5.82* ±4.22* | 5.87* ±4.20* | 12.1 ±4.70 |
|  |  | 12.0\| 10mins \|1.2/min | 6 | 33.33 | 34.5 | 59.6 | 4 | 30.6 ±18.1 | {0.18} (0.10-0.19) | 7.52* ±3.82* | 7.58* ±3.82* | 9.5 ±4.00 |
|  |  | 17.0\| 10mins \|1.7/min | 5 | 83.33 | 43 | 74.2 | 4 | 72.1 ±47.1 | {0.16} (0.16-0.19) | 14.03* ±7.55* | NR | NR |
|  |  | 21.5\| 10mins \|2.15/min | 6 | 83.33 | 40 | 80.6 | 4 | 62.7 ±25.8 | {0.16} (0.16-0.18) | 13.92* ±3.85* | 13.95* ±3.85* | 12.1 ±5.20 |
| Strassman et al 1996 | DMT (Intravenous) | 21.0 [30s bolus] | 13 | 69.23 | 35.5 | NR | 2 | 62.5 ±6.60 | NR | NR | NR | NR |
|  |  |  |  |  |  |  |  |  |  |  |  |  |
|  |  |  |  |  |  |  |  |  |  |  |  |  |
| **Study Reference** | **Compound (Route)** | **Dose  (mg)** | **N Analysed** | **Male %** | **Mean Age (Years)** | **Mean Weight (Kg)** | **PK Session (Hours)** | **Cmax (ng/mL)** | **Tmax (Hours)** | **AUC 0-t (ng*h/mL)** | **AUC 0-inf (ng*h/mL)** | **T 1/2  (Hours)** |
| van der Heijden et al 2025 | DMT (Intravenous) | 39.3\| 360mins \|0.105/min | 8 | 62.50 | 32 | 71.6 | 10 | 4.6 ±2.90 | {0.90} (0.00-5.90) | 14.2 ±6.80 | 13.5 ±7.10 | 0.2 ±0.10 |
|  |  | 197\| 360mins \|0.525/min | 8 | 50.00 | 26 | 70.5 | 10 | 25.3 ±24.1 | {0.90} (0.00-5.00) | 85.9 ±21.6 | 97.8 ±16.8 | 0.2 ±0.00 |
|  |  | 289\| 360mins \|0.787/min | 7 | 42.90 | 23.4 | 70.7 | 10 | 35.9 ±34.0 | {1.90} (0.80-5.10) | 157.6 ±55.3 | 157.8 ±55.3 | 0.3 ±0.10 |
| Vogt et al 2023 | DMT (Intravenous) | 54.0\| 90mins \|0.600mg/min | 27 | 44.45 | 33.0 | NR | 2.5 | 24 [21-28] | 1.20  [1.12–1.30] | 22.8*  [19.5–26.7]* | 22.9*  [19.6–26.8]* | 0.25  [0.22–0.27] |
|  |  | 90.0\| 90mins \|1.00mg/min |  |  |  |  |  | 39 [33-45] | 1.22  [1.07–1.38] | 38.3*  [32.7–44.9]* | 38.4*  [32.8–45.0]* | 0.25  [0.23–0.27] |
|  |  | 15mg [45s bolus]  + 54.0\| 90mins \|0.600mg/min |  |  |  |  |  | 29 [26-33] | 0.042  [0.035–0.048] | 31.7*  [27.5–36.4]* | 31.7*  [27.6–36.5]* | 0.23  [0.22–0.27] |
|  |  | 25mg [45s bolus]  + 90.0\| 90mins \|1.00mg/min |  |  |  |  |  | 61 [51-73] | 0.048  [0.040–0.060] | 53.6*  [48.1–59.7]* | 53.7*  [48.2–59.8]* | 0.27  [0.23–0.28] |
| Callaway et al 1999 | DMT (Oral) | 35.5 | 12 | 100 | 35.9 | 74.2 | 24 | 15.8 ±4.40 | 1.79 ±0.54 | NR | 5.6* ±4.53* | 4.32 ±3.45 |
| Egger et al 2025 | DMT (Oral) | 90.0 | 8 | 56.25 | 35 | NR | 9 | 4.5 ±2.40 | 1.14 ±0.26 | 3.6 ±1.80 | 5 ±1.70 | 0.52 ±0.42 |
| Lanaro et al 2021 | DMT (Oral) | 217 | 14 | 64.29 | 39.5 | NR | 24 | 7.69 | 1.3 | 20.2 | NR | 3.51 NR |
| Riba et al 2003 | DMT (Oral) | 39.8 | 14 | 83.33 | 25.7 | 66.47 | 8 | 12.1 ±9.09 | {1.50} (1.00–2.50) | 18.8 ±10.7 | 21.6 ±9.93 | 1.07 ±0.58 |
|  |  | 57.4 | 15 |  |  |  |  | 17.4  ±10.5 | {1.50} (1.00-4.00) | 33.2 ±14.7 | 38.3 ±17.5 | 1.06 ±0.77 |
| Arikci et al 2025 | LSD (Oral) | 0.083 | 20 | 50.00 | 37 | 70 | 24 | 1.76 [1.53-2.02] | 2 [1.70-2.40] | 13.5* [11.40-16.00]* | 14.1 [11.90-16.80] | 4 [3.60-4.40] |
| Becker et al 2023 | LSD (Oral) | 0.093 | 24 | 50.00 | 34 | 71 | 12 | 2.13 ±0.72 | 2 ±0.80 | 13.8 ±5.90 | 17 ±9.00 | 4.2 ±1.60 |
| Becker et al 2025 | LSD (Oral) | 0.100 | 23 | 52.17 | 31 | 68 | 24 | 2 [1.70-2.30] | 1.8 [1.40-2.30] | NR | 15 [13.0-17.0] | 3.5 [3.20-3.80] |
| Dolder et al 2015 | LSD (Oral) | 0.200 | 16 | 50.00 | 28.6 | NR | 24 | 4.3 [3.80-4.90] | 1.5 [0.50-4.00] | 26 [22.0-30.0] (90%CI) | 28 [24.0-33.0] (90%CI) | 3.6 ±0.90 |
| **Study Reference** | **Compound (Route)** | **Dose  (mg)** | **N Analysed** | **Male %** | **Mean Age (Years)** | **Mean Weight (Kg)** | **PK Session (Hours)** | **Cmax (ng/mL)** | **Tmax (Hours)** | **AUC 0-t (ng*h/mL)** | **AUC 0-inf (ng*h/mL)** | **T 1/2  (Hours)** |
| Family et al 2020 | LSD (Oral) | 0.010 | 7 | 50.00 | 63.17 | NR | 12 | 0.305 ±0.068 | 0.5 ±0.00 | 0.891* ±0.104* | 3.195 ±0.454 | 8.25 ±7.50 |
|  |  | 0.020 | 8 | 75.00 | 61.58 | NR | 12 | 0.44 ±0.079 | 0.5 ±0.00 | 1.407* ±0.183* | 4.466 ±0.948 | 8.25 ±7.50 |
| Family et al 2022 | LSD (Oral) | 0.050 | 3 | 100 | 28.3 | NR | 24 | 1.09 ±0.219 | 2 ±0.00 | 4.337* ±1.723* | NR | 2.8 ±0.30 |
|  |  | 0.075 | 7 | 85.71 | 28.3 | NR | 24 | 1.71 ±0.417 | 1.2 ±0.57 | 7.577* ±2.880* |  | 3.2 ±0.70 |
|  |  | 0.100 | 3 | 100 | 32 | NR | 24 | 3.03 ±0.554 | 1.7 ±0.60 | 17.723* ±0.868* |  | 4.3 ±1.30 |
| Holze et al 2019 | LSD (Oral) | 0.096 | 27 | 48.15 | 28 | 71 | 12 | 1.7 [1.60-2.00] | 1.7 [1.60-2.00] | NR | 13 [12.0-16.0] | 3.6 [3.30-4.30] |
| Holze et al 2021-A | LSD (Oral) | 0.005 | 13 | 52.17 | 23 | 70 | 6 | 0.151 [0.127-0.181] | 1.1 [0.91-1.40] | NR | 0.8 [0.658-0.968] | 2.5 (1.50-3.90) |
|  |  | 0.010 | 18 |  |  |  |  | 0.279 [0.240-0.320] | 1 [0.71-1.40] |  | 1.544 [1.273-1.872] | 2.7 [2.30-3.20] |
|  |  | 0.020 | 15 |  |  |  |  | 0.5 [0.413-0.607] | 1.1 [0.77-1.50] |  | 2.912 [2.271-3.734] | 2.9 [2.40-3.50] |
| Holze et al 2021-B | LSD (Oral) | 0.025 | 16 | 50.00 | 29 | 69 | 24 | 0.49 [0.410-0.580] | 1.2 [0.90-1.70] | NR | 3.5 [2.70-4.50] | 3.6 [2.90-4.40] |
|  |  | 0.050 |  |  |  |  |  | 1.1 [0.990-1.20] | 1.2 [0.95-1.60] |  | 7.4 [6.20-8.90] | 3.6 [3.00-4.20] |
|  |  | 0.100 |  |  |  |  |  | 2 [1.90-2.20] | 1.4 [1.20-1.70] |  | 15 [12.0-18.0] | 3.9 [3.20-4.70] |
|  |  | 0.200 |  |  |  |  |  | 3.9 [3.50-4.30] | 1.5 [1.30-1.90] |  | 31 [25.0-38.0] | 4.1 [3.40-4.90] |
| Holze et al 2022 | LSD (Oral) | 0.100 | 28 | 50.00 | 35 | NR | 24 | 1.9 [1.50-2.00] | 1.6 [1.30-1.70] | 13 [11.0-14.0] | 14 [11.0-15.0] | 4.3 [3.80-4.60] |
|  |  | 0.200 |  |  |  |  |  | 3.4 [2.90-3.70] | 1.6 [1.10-1.70] | 26 [20.0-28.0] | 27 [21.0-29.0] | 4 [3.50-4.30] |
| Holze et al 2023 | LSD (Oral) | 0.085 | 28 | 50.00 | 35 | NR | 24 | 1.8 [1.60-2.00] | 1.7 [1.50-1.90] | NR | 13 [11.0-16.0] | 3.7 [3.40-4.10] |
|  |  | 0.170 |  |  |  |  |  | 3.4 [3.00-3.80] | 1.6 [1.30-1.90] |  | 27 [22.0-32.0] | 4 [3.60-4.40] |
| **Study Reference** | **Compound (Route)** | **Dose  (mg)** | **N Analysed** | **Male %** | **Mean Age (Years)** | **Mean Weight (Kg)** | **PK Session (Hours)** | **Cmax (ng/mL)** | **Tmax (Hours)** | **AUC 0-t (ng*h/mL)** | **AUC 0-inf (ng*h/mL)** | **T 1/2  (Hours)** |
| Ley et al 2023 | LSD (Oral) | 0.093 | 32 | 50.00 | 29 | NR | 24 | 2.1 [1.90-2.40] | 1.4 [1.3-1.8] | 14 [13.0-17.0] | 14 [13.0-17.0] | 3.5 [3.30-3.80] |
| Morse et al 2025 | LSD (Oral) | 0.010 | 39 | 100 | 37.4 | 85.1 | 6 | 0.2 ±0.130 | 1.51 ±0.66 | NR | NR | 3.09 ±0.37 |
| Arikci et al 2025 | LSD Tartrate (Intravenous) | 0.080\|0.009/min | 20 | 50.00 | 37 | 70 | 24 | 5.94 [4.95-7.12] | 0.16 [0.15-0.17] | 16.8 [14.60-19.30] | 17.2 [14.90-20.00] | 3.8 [3.60-4.10] |
| Klaiber et al 2024 | Mescaline (Oral) | 100 | 16 | 50.00 | 33 | NR | 30 | 298 [268-332] | 1.6 [1.30-2.00] | 1767 [1660-1880] | 1805 [1700-1916] | 3.5 [3.20-3.80] |
|  |  | 200 |  |  |  |  |  | 568 [522-619] | 1.9 [1.50-2.30] | 3588 [3342-3853] | 3636 [3393-3898] | 3.7 [3.40-4.00] |
|  |  | 400 |  |  |  |  |  | 1034 [944-1132] | 2.3 [1.80-2.90] | 7435 [6791-8140] | 7493 [6844-8204] | 3.7 [3.40-3.90] |
|  |  | 800 |  |  |  |  |  | 1721 [1482-1998] | 2.2 [1.50-3.20] | 13047 [11,119-15,310] | 13144 [11,190-15,439] | 3.7 [3.50-4.00] |
| Ley et al 2023 | Mescaline (Oral) | 300 | 16 | 50.00 | 29 | NR | 24 | 858 [769-992] | 2.3 [1.90-2.90] | 6461 [6028-7022] | 6558 [6117-7132] | 3.6 [3.50-3.80] |
|  |  | 500 |  |  |  |  |  | 1217 [1084-1426] | 2.3 [1.90-3.00] | 8974 [8209-10,178] | 9115 [8344-10315] | 3.6 [3.30-3.80] |
| Hasler et al 1997 | Psilocybin/Psilocin (Intravenous) | 1.00 [30s bolus] | 6 | 88.89 | 30 | 65.16 | 2 | 12.9 ±5.60 | 0.03 ±0.02 | NR | 4.00 ±0.92 | 1.24 ±0.33 |
| Becker et al 2022 | Psilocybin/Psilocin (Oral) | 25.0 | 23 | 52.17 | 34 | 70 | 7 | 20 ±5.40 | 2 (1.00-4.00) | 73 ±17.0 | 83 ±21.0 | 1.8 ±0.30 |
| Brown et al 2017 | Psilocybin/Psilocin (Oral) | 23.4 | 12 | 83.00 | 43 | 78.1 | 24 | {16} 14.5-17.2☨ | 2.03 [1.15-2.07] | 140* (102-175)* | NR | 3 ±1.10 |
|  |  | 35.1 | 11 |  |  |  |  | {26} 22.7-35.1☨ | 2.03 (1.30-3.00) | 213* (150-261) |  | 3 ±1.10 |
|  |  | 46.9 | 10 |  |  |  |  | {37.6} 27.7-43.2☨ | 2.05 [1.55-2.08] | 267* (201-356)* |  | 3 ±1.10 |
| Hasler et al 1997 | Psilocybin/Psilocin (Oral) | 1.84 | 6 | 100 | 33 | 74.1 | 7 | 8.2 ±2.80 | 1.75 ±0.62 | NR | 32.72* ±10.98* | 2.72 ±1.06 |
| Holze et al 2022 | Psilocybin/Psilocin (Oral) | 0.015 | 28 | 50.00 | 35 | NR | 24 | 13 [12.0-15.0] | 2.3 [1.90-2.50] | 59 [52.0-63.0] | 61 [54.0-65.0] | 2.4 [2.30-2.60] |
|  |  | 0.030 |  |  |  |  |  | 25 [21.0-27.0] | 2.5 [2.00-2.70] | 119 [105-128] | 131 [107-130] | 2.7 [2.50-2.80] |
| Ley et al 2023 | Psilocybin/Psilocin (Oral) | 18.4 | 32 | 50.00 | 29 | NR | 24 | 17 [15.0-19.0] | 2.1 [1.90-2.40] | 84 [76.0-92.0] | 85 [78.0-94.0] | 2.3 [2.10-2.40] |
| Lindenblatt 1998 | Psilocybin/Psilocin (Oral) | 14.0 | 7 | NR | NR | NR | 7 | 11.3 ±5.53 | 1.36 ±10.7 | 30.2 ±7.80 | NR | NR |
| **Study Reference** | **Compound (Route)** | **Dose  (mg)** | **N Analysed** | **Male %** | **Mean Age (Years)** | **Mean Weight (Kg)** | **PK Session (Hours)** | **Cmax (ng/mL)** | **Tmax (Hours)** | **AUC 0-t (ng*h/mL)** | **AUC 0-inf (ng*h/mL)** | **T 1/2  (Hours)** |
| Mallaroni et al 2023 | Psilocybin/Psilocin (Oral) | 15.0 | 21 | 50.00 | 25 | 69.94 | 7 | 10.81 (5.26-25.5) | 3.71 (2.00-5.00) | 33 (21.7–71.6) | NR | 2.24 (1.44–3.81) |
| Mason et al 2020 | Psilocybin/Psilocin (Oral) | 11.9 | 30 | 60.00 | 22.73 | NR | 6 | 15.60 | 1.30 | NR | NR | NR |
| Viktorin et al 2022 | Psilocybin/Psilocin (Oral) | 17.8 | 12 | NR | 36 | NR | 6 | 13.6 ±4.61 | 2.00 | NR | NR | NR |

* See compound specific summary table in supplementary materials for details on converted units; {Median}; ☨ Inter-quartile range; {Median}; (95%CI); [Range]

**Supplementary Material 4a -** Summary Table of Oral LSD Pharmacokinetic Parameters Sorted by Dose

| **Study Reference** | **Dose  (mg)** | **N Analysed** | **PK Session**  **(Hours)** | **Cmax (ng/mL)** | **Tmax (Hours)** | **AUC 0-t (ng*h/mL)** | **AUC 0-inf (ng*h/mL)** | **T 1/2  (Hours)** | **Clearance (L/h)** | **Volume Distribution Vz/F (L)** |
| --- | --- | --- | --- | --- | --- | --- | --- | --- | --- | --- |
| Holze et al 2021-A | 0.005 | 13 | 6 | 0.151 [0.127-0.181] | 1.1 [0.91-1.40] | NR | 0.8 [0.658-0.968] | 2.5 (1.50-3.90) | 6.3 [5.20-7.60] | 23 [19.0-28.0] |
| Family et al 2020 | 0.010 | 7 | 12 | 0.305 ±0.068 | 0.5 ±0.00 | 0.891* ±0.104* | 3.195 ±0.454 | 8.25 ±7.50 | NR | NR |
| Holze et al 2021-A | 0.010 | 18 | 6 | 0.279 [0.240-0.320] | 1 [0.71-1.40] | NR | 1.544 [1.273-1.872] | 2.7 [2.30-3.20] | 6.5 [5.30-7.90] | 25 [21.0-30.0] |
| Morse et al 2025 | 0.010 | 39 | 6 | 0.2 ±0.130 | 1.51 ±0.66 | NR | NR | 3.09 ±0.37 | 7.78☨ [6.75-8.77]☨ | 40 [36.6-43.8] |
| Family et al 2020 | 0.020 | 8 | 12 | 0.44 ±0.079 | 0.5 ±0.00 | 1.407* ±0.183* | 4.466 ±0.948 | 8.25 ±7.50 | NR | NR |
| Holze et al 2021-A | 0.020 | 15 | 6 | 0.5 [0.413-0.607] | 1.1 [0.77-1.50] | NR | 2.912 [2.271-3.734] | 2.9 [2.40-3.50] | 6.9 [5.40-8.08] | 29 [23.0-36.0] |
| Holze et al 2021-B | 0.025 | 16 | 24 | 0.49 [0.410-0.580] | 1.2 [0.90-1.70] | NR | 3.5 [2.70-4.50] | 3.6 [2.90-4.40] | 7.2 [5.60-9.30] | 38 [30.0-47.0] |
| Family et al 2022 | 0.050 | 3 | 24 | 1.09 ±0.219 | 2 ±0.00 | 4.337* ±1.723* | NR | 2.8 ±0.30 | NR | NR |
| Holze et al 2021-B | 0.050 | 16 | 24 | 1.1 [0.990-1.20] | 1.2 [0.95-1.60] | NR | 7.4 [6.20-8.90] | 3.6 [3.00-4.20] | 6.7 [5.60-8.00] | 35 [31.0-38.0] |
| Family et al 2022 | 0.075 | 7 | 24 | 1.71 ±0.417 | 1.2 ±0.57 | 7.577* ±2.880* | NR | 3.2 ±0.70 | NR | NR |
| Arikci et al 2025 | 0.083 | 20 | 24 | 1.76 [1.53-2.02] | 2 [1.70-2.40] | 13.5* [11.40-16.00]* | 14.1 [11.90-16.80] | 4 [3.60-4.40] | 5.9 [4.90-7.00] | 34 [30.0-39.0] |
| Holze et al 2023 | 0.085 | 28 | 24 | 1.8 [1.60-2.00] | 1.7 [1.50-1.90] | NR | 13 [11.0-16.0] | 3.7 [3.40-4.10] | 7.4 [6.30-8.50] | 38 [33.0-42.0] |
| Becker et al 2023 | 0.093 | 24 | 12 | 2.13 ±0.72 | 2 ±0.80 | 13.8 ±5.90 | 17 ±9.00 | 4.2 ±1.60 | 7.2 ±2.90 | 39 ±10.0 |
| Ley et al 2023 | 0.093 | 32 | 24 | 2.1 [1.90-2.40] | 1.4 [1.3-1.8] | 14 [13.0-17.0] | 14 [13.0-17.0] | 3.5 [3.30-3.80] | 6.5 [6.00-8.20] | 33 [30.0-38.0] |
| **Study Reference** | **Dose  (mg)** | **N Analysed** | **PK Session**  **(Hours)** | **Cmax (ng/mL)** | **Tmax (Hours)** | **AUC 0-t (ng*h/mL)** | **AUC 0-inf (ng*h/mL)** | **T 1/2  (Hours)** | **Clearance (L/h)** | **Volume Distribution Vz/F (L)** |
| Holze et al 2019 | 0.096 | 27 | 12 | 1.7 [1.60-2.00] | 1.7 [1.60-2.00] | NR | 13 [12.0-16.0] | 3.6 [3.30-4.30] | 7.5 [6.90-9.00] | 39 [36.0-47.0] |
| Becker et al 2025 | 0.100 | 23 | 24 | 2.0 [1.70-2.30] | 1.8 [1.40-2.30] | NR | 15 [13.0-17.0] | 3.5 [3.20-3.80] | 6.8 [5.90-8.00] | 34 [31.0-38.0] |
| Family et al 2022 | 0.100 | 3 | 24 | 3.03 ±0.554 | 1.7 ±0.60 | 17.723* ±0.868* | NR | 4.3 ±1.30 | NR | NR |
| Holze et al 2021-B | 0.100 | 16 | 24 | 2.0 [1.90-2.20] | 1.4 [1.20-1.70] | NR | 15 [12.0-18.0] | 3.9 [3.20-4.70] | 6.6 [5.40-8.00] | 37 [33.0-42.0] |
| Holze et al 2022 | 0.100 | 28 | 24 | 1.9 [1.50-2.00] | 1.6 [1.30-1.70] | 13 [11.0-14.0] | 14 [11.0-15.0] | 4.3 [3.80-4.60] | 7.1 [5.60-7.70] | 41 [35.0-45.0] |
| Holze et al 2023 | 0.170 | 28 | 24 | 3.4 [3.00-3.80] | 1.6 [1.30-1.90] | NR | 27 [22.0-32.0] | 4 [3.60-4.40] | 7.5 [6.40-8.70] | 42 [36.0-48.0] |
| Dolder et al 2015 | 0.200 | 16 | 24 | 4.3 [3.80-4.90] | 1.5 [0.50-4.00] | 26 [22.0-30.0] (90%CI) | 28 [24.0-33.0] (90%CI) | 3.6 ±0.90 | 4.74ª  ±2.16ª | NR |
| Holze et al 2021-B | 0.200 | 16 | 24 | 3.9 [3.50-4.30] | 1.5 [1.30-1.90] | NR | 31 [25.0-38.0] | 4.1 [3.40-4.90] | 6.5 [5.30-8.00] | 39 [35.0-43.0] |
| Holze et al 2022 | 0.200 | 28 | 24 | 3.4 [2.90-3.70] | 1.6 [1.10-1.70] | 26 [20.0-28.0] | 27 [21.0-29.0] | 4 [3.50-4.30] | 7.5 [5.80-8.20] | 41 [34.0-44.0] |

* Converted from pg*hour/mL; ª Converted from (mL/min); ☨ Converted from L/h/70 kg; {Median}; (95%CI); [Range]

**Supplementary Material 4b –** Scatter plots with regression lines of pharmacokinetic parameters for Oral LSD

Pharmacokinetic parameters (Cmax, Tmax, T½, AUC, clearance, volume distribution) are plotted against dose as circular scatter points. Lines represent simple linear regression fits weighted by trial arm sample size (n). In subplot a (Tmax & T½), Tmax is shown with blue circles and a navy regression line, while T½ is shown with orange circles and a red regression line. In subplots b–f, pharmacokinetic parameters are shown with light blue circles and orange regression lines.

**Supplementary Material 5a -**Summary Table of Oral Psilocybin/Psilocin Pharmacokinetic Parameters Sorted by Dose

| **Study Reference** | **Dose  (mg)** | **N Analysed** | **PK Session (Hours)** | **Cmax (ng/mL)** | **Tmax (Hours)** | **AUC 0-t (ng*h/mL)** | **AUC 0-inf (ng*h/mL)** | **T 1/2  (Hours)** | **Clearance (L/h)** | **Volume Distribution Vz/F (L)** |
| --- | --- | --- | --- | --- | --- | --- | --- | --- | --- | --- |
| Mason et al 2020 | 11.9 | 30 | 6 | 15.6 | 1.3 | NR | NR | NR | NR | NR |
| Lindenblatt 1998 | 14.0 | 7 | 7 | 11.3 ±5.53 | 1.36 ±10.7 | 30.2 ±7.80 | NR | NR | NR | NR |
| Hasler et al 1997 | 15.0 | 6 | 7 | 8.2 ±2.80 | 1.75 ±0.62 | NR | 32.72ª ±10.98ª | 2.72 ±1.06 | NR | NR |
| Holze et al 2022 | 15.0 | 28 | 24 | 13 [12.0-15.0] | 2.3 [1.90-2.50] | 59 [52.0-63.0] | 61 [54.0-65.0] | 2.4 [2.30-2.60] | 262 [230-279] | 925 [792-992] |
| Mallaroni et al 2023 | 15.0 | 21 | 7 | 10.81 (5.26-25.5) | 3.71 (2.00-5.00) | 33 (21.7–71.6) | NR | 2.24 (1.44–3.81) | NR | NR |
| Viktorin et al 2022 | 17.8 | 12 | 6 | 13.6 ±4.61 | 2 | NR | NR | NR | NR | NR |
| Ley et al 2023 | 18.4 | 32 | 24 | 17 [15.0-19.0] | 2.1 [1.90-2.40] | 84 [76.0-92.0] | 85 [78.0-94.0] | 2.3 [2.10-2.40] | 155 [145-177] | 505 [467-602] |
| Brown et al 2017 | 23.4 | 12 | 24 | {16} 14.5-17.2 IQR | 2.03 [1.15-2.07] | 140* (102-175)* | NR | 3 ±1.10 | 164 ±23.2 | 298 ±20.2 |
| Becker et al 2022 | 25.0 | 23 | 7 | 20 ±5.40 | 2 (1.00-4.00) | 73 ±17.0 | 83 ±21.0 | 1.8 ±0.30 | NR | NR |
| Holze et al 2022 | 30.0 | 28 | 24 | 25 [21.0-27.0] | 2.5 [2.00-2.70] | 119 [105-128] | 131 [107-130] | 2.7 [2.50-2.80] | 263 [231-281] | 1016 [883-1090] |
| Brown et al 2017 | 35.1 | 11 | 24 | {26} 22.7-35.1☨ | 2.03 (1.30-3.00) | 213* (150-261) | NR | 3 ±1.10 | 164 ±23.2 | 298 ±20.2 |
| Brown et al 2017 | 46.9 | 10 | 24 | {37.6} 27.7-43.2☨ | 2.05 [1.55-2.08] | 267* (201-356)* | NR | 3 ±1.10 | 164 ±23.2 | 298 ±20.2 |

***** Converted from µg*h/L; ª Converted from ng*min/mL; ☨ Inter-quartile range; {Median}; (95%CI); [Range]

**Supplementary Material 5b –** Scatter plots with regression lines of pharmacokinetic parameters for Oral Psilocybin/Psilocin

Pharmacokinetic parameters (Cmax, Tmax, T½, AUC, clearance, volume distribution) are plotted against dose as circular scatter points. Lines represent simple linear regression fits weighted by trial arm sample size (n). In subplot a (Tmax & T½), Tmax is shown with blue circles and a navy regression line, while T½ is shown with orange circles and a red regression line. In subplots b–f, pharmacokinetic parameters are shown with light blue circles and orange regression lines.

**Supplementary Material 6a -** Summary Table of Intravenous DMT Pharmacokinetic Parameters

| **Study Reference** | **Dose (mg) \| Infusion rate** | **N Analysed** | **PK Session (Hours)** | **Cmax (ng/mL)** | **Tmax (Hours)** | **AUC 0-t (ng*h/mL)** | **AUC 0-inf (ng*h/mL)** | **T 1/2  (Hours)** | **Clearance (L/h)** | **Volume Distribution Vz/F (L)** |
| --- | --- | --- | --- | --- | --- | --- | --- | --- | --- | --- |
| Erne et al 2025 | 72.0\| 120mins \|0.6/min | 22 | 3 | 26 [22.0-31.0] | 1.43 [1.23-1.65] | NR | 35.88* [29.45-43.73]* | 0.32  (0.27–0.38) | 2040ª [1680-2460]ª | 912 [704-1183] |
|  | 144\| 120mins \|1.2/min |  |  | 51 [42.0-61.0] | 1.7 [1.50-1.93] |  | 67.58* [55.37-82.50]* | 0.30  (0.28–0.33) | 2160ª [1740-2640]ª | 941 [743-1192] |
|  | 216\| 120mins \|1.8/min |  |  | 78 [63.0-96.0] | 1.6 [1.42-1.80] |  | 105.7* [84.05-133.0]* | 0.30  (0.27–0.32) | 2040ª [1620-2580]ª | 862 [663-1121] |
|  | 288\| 120mins \|2.4/min |  |  | 105 [85.0-128] | 1.6 [1.35-1.90] |  | 151.63* [123.2-186.7]* | 0.30  (0.28–0.33) | 1920ª [1560-2340]ª | 832 [654-1058] |
| Good et al 2023 | 9.00\| 10mins \|0.9/min | 5 | 4 | 20.8 ±12.9 | {0.16} (0.11-0.18) | 5.82* ±4.22* | 5.87* ±4.20* | 0.20  ±0.08 | 2760ª ±2616ª | 611 ±308 |
|  | 12.0\| 10mins \|1.2/min | 6 |  | 30.6 ±18.1 | {0.18} (0.10-0.19) | 7.52* ±3.82* | 7.58* ±3.82* | 0.16  ±0.07 | 1944ª ±876ª | 425 ±214 |
|  | 17.0\| 10mins \|1.7/min | 5 |  | 72.1 ±47.1 | {0.16} (0.16-0.19) | 14.03* ±7.55* | NR | NR | NR | NR |
|  | 21.5\| 10mins \|2.15/min | 6 |  | 62.7 ±25.8 | {0.16} (0.16-0.18) | 13.92* ±3.85* | 13.95* ±3.85* | 0.20  ±0.09 | 1674ª ±582ª | 456 ±157 |
| Strassman et al 1996 | 21.0 [30s bolus] | 13 | 2 | 62.5 ±6.60 | NR | NR | NR | NR | NR | NR |
| van der Heijden et al 2025 | 39.3\| 360mins \|0.105/min | 8 | 10 | 4.6 ±2.90 | {0.90} (0.00-5.90) | 14.2 ±6.80 | 13.5 ±7.10 | 0.2 ±0.10 | 2086 ±706 | 616 ±291 |
|  | 197\| 360mins \|0.525/min | 8 |  | 25.3 ±24.1 | {0.90} (0.00-5.00) | 85.9 ±21.6 | 97.8 ±16.8 | 0.2 ±0.00 | 1273 ±226.6 | 431 ±109 |
|  | 289\| 360mins \|0.787/min | 7 |  | 35.9 ±34.0 | {1.90} (0.80-5.10) | 157.6 ±55.3 | 157.8 ±55.3 | 0.3 ±0.10 | 1272 ±485.2 | 450 ±199 |
| Vogt et al 2023 | 54.0\| 90mins \|0.600mg/min | 27 | 2.5 | 24 [21-28] | 1.20  [1.12–1.30] | 22.8*  [19.5–26.7]* | 22.9*  [19.6–26.8]* | 0.25  [0.22–0.27] | 2340ª [2040–2760]ª | 820  [671–1004] |
|  | 90.0\| 90mins \|1.00mg/min |  |  | 39 [33-45] | 1.22  [1.07–1.38] | 38.3*  [32.7–44.9]* | 38.4*  [32.8–45.0]* | 0.25  [0.23–0.27] | 2340ª [1980–2760]ª | 831  [683–1012] |
|  | 15mg [45s bolus]  + 54.0\| 90mins \|0.600mg/min |  |  | 29 [26-33] | 0.042  [0.035–0.048] | 31.7*  [27.5–36.4]* | 31.7*  [27.6–36.5]* | 0.23  [0.22–0.27] | 2160ª [1920–2520]ª | 746  [639–871] |
|  | 25mg [45s bolus]  + 90.0\| 90mins \|1.00mg/min |  |  | 61 [51-73] | 0.048  [0.040–0.060] | 53.6*  [48.1–59.7]* | 53.7*  [48.2–59.8]* | 0.27  [0.23–0.28] | 2160ª [1920–2400]ª | 799  [693–922] |

* Converted from ng*min/mL; ª Converted from L/min; {Median}; (95%CI); [Range]

**Supplementary Material 6b –** Scatter plots with regression lines of pharmacokinetic parameters for Intravenous DMT

Pharmacokinetic parameters (Cmax, Tmax, T½, AUC, clearance, volume distribution) are plotted against dose as circular scatter points. Lines represent simple linear regression fits weighted by trial arm sample size (n). In subplot a (Tmax & T½), Tmax is shown with blue circles and a navy regression line, while T½ is shown with orange circles and a red regression line. In subplots b–f, pharmacokinetic parameters are shown with light blue circles and orange regression lines.

**Supplementary Material 7a -** Summary Table of Oral DMT Pharmacokinetic Parameters Sorted by Dose

| **Study Reference** | **Dose  (mg)** | **N Analysed** | **PK Session (Hours)** | **Cmax (ng/mL)** | **Tmax (Hours)** | **AUC 0-t (ng*h/mL)** | **AUC 0-inf (ng*h/mL)** | **T 1/2  (Hours)** | **Clearance (L/h)** | **Volume Distribution Vz/F (L)** |
| --- | --- | --- | --- | --- | --- | --- | --- | --- | --- | --- |
| Callaway et al 1999 | 35.5 | 12 | 24 | 15.8 ±4.40 | 1.79 ±0.54 | NR | 5.6* ±4.53* | 4.32 ±3.45 | 986.8ª ±578.5ª | 54.8⌔ ±14.8⌔ |
| Riba et al 2003 | 39.8 | 14 | 8 | 12.1 ±9.09 | {1.50} (1.00–2.50) | 18.8 ±10.7 | 21.6 ±9.93 | 1.07 ±0.58 | 2281 ±1055 | 3510 ±2158 |
| Riba et al 2003 | 57.4 | 15 | 8 | 17.4  ±10.5 | {1.50} (1.00-4.00) | 33.2 ±14.7 | 38.3 ±17.5 | 1.06 ±0.77 | 1813 ±804 | 2506 ±1529 |
| Egger et al 2025 | 90.0 | 8 | 9 | 4.5 ±2.40 | 1.14 ±0.26 | 3.6 ±1.80 | 5 ±1.70 | 0.52 ±0.42 | 6720☨ ±2988☨ | 3351 ±1818 |
| Lanaro et al 2021 | 217 | 14 | 24 | 7.69 | 1.30 | 20.20 | NR | 3.51 | NR | NR |

* Reported in mg*min/mL as per original paper; ª Converted from mL/min/kg; ☨ Converted from L/min; ⌔ Reported in Vss:F (l:kg) as per original paper; {Median}; (95%CI)

**Supplementary Material 7b –** Scatter plots with regression lines of pharmacokinetic parameters for Oral DMT

Pharmacokinetic parameters (Cmax, Tmax, T½, AUC, clearance, volume distribution) are plotted against dose as circular scatter points. Lines represent simple linear regression fits weighted by trial arm sample size (n). In subplot a (Tmax & T½), Tmax is shown with blue circles and a navy regression line, while T½ is shown with orange circles and a red regression line. In subplots b–f, pharmacokinetic parameters are shown with light blue circles and orange regression lines. Note the low number of studies which limits explanatory value.

**Supplementary Material 8a -** Summary Table of Oral Mescaline Pharmacokinetic Parameters Sorted by Dose

| **Study Reference** | **Dose  (mg)** | **N Analysed** | **PK Session (Hours)** | **Cmax (ng/mL)** | **Tmax (Hours)** | **AUC 0-t (ng*h/mL)** | **AUC 0-inf (ng*h/mL)** | **T 1/2  (Hours)** | **Clearance (L/h)** | **Volume Distribution Vz/F (L)** |
| --- | --- | --- | --- | --- | --- | --- | --- | --- | --- | --- |
| Klaiber et al 2024 | 100 | 16 | 30 | 298 [268-332] | 1.6 [1.30-2.00] | 1767 [1660-1880] | 1805 [1700-1916] | 3.5 [3.20-3.80] | 55 [52.0-59.0] | 281 [254-312] |
| Klaiber et al 2024 | 200 | 16 | 30 | 568 [522-619] | 1.9 [1.50-2.30] | 3588 [3342-3853] | 3636 [3393-3898] | 3.7 [3.40-4.00] | 55 [51.0-59.0] | 292 [266-321] |
| Ley et al 2023 | 300 | 16 | 24 | 858 [769-992] | 2.3 [1.90-2.90] | 6461 [6028-7022] | 6558 [6117-7132] | 3.6 [3.50-3.80] | 37 [34.0-40.0] | 188 [173-209] |
| Klaiber et al 2024 | 400 | 16 | 30 | 1034 [944-1132] | 2.3 [1.80-2.90] | 7435 [6791-8140] | 7493 [6844-8204] | 3.7 [3.40-3.90] | 53 [49.0-58.0] | 283 [258-311] |
| Ley et al 2023 | 500 | 16 | 24 | 1217 [1084-1426] | 2.3 [1.90-3.00] | 8974 [8209-10,178] | 9115 [8344-10315] | 3.6 [3.30-3.80] | 45 [39.0-53.0] | 213 [185-257] |
| Klaiber et al 2024 | 800 | 16 | 30 | 1721 [1482-1998] | 2.2 [1.50-3.20] | 13047 [11,119-15,310] | 13144 [11,190-15,439] | 3.7 [3.50-4.00] | 61 [52.0-72.0] | 328 [282-381] |

[Range]

**Supplementary Material 8b –** Scatter plots with regression lines of pharmacokinetic parameters for Oral Mescaline

Pharmacokinetic parameters (Cmax, Tmax, T½, AUC, clearance, volume distribution) are plotted against dose as circular scatter points. Lines represent simple linear regression fits weighted by trial arm sample size (n). In subplot a (Tmax & T½), Tmax is shown with blue circles and a navy regression line, while T½ is shown with orange circles and a red regression line. In subplots b–f, pharmacokinetic parameters are shown with light blue circles and orange regression lines.

**Supplementary Material 9a-** Summary Table of 5-MEO-DMT Pharmacokinetic Parameters Sorted by Dose

| **Study Reference** | **Compound/Route** | **Dose  (mg)** | **N Analysed** | **PK Session (Hours)** | **Cmax (ng/mL)** | **Tmax (Hours)** | **AUC 0-t (ng*h/mL)** | **AUC 0-inf (ng*h/mL)** | **T 1/2  (Hours)** | **Clearance (L/h)** | **Volume Distribution Vz/F (L)** |
| --- | --- | --- | --- | --- | --- | --- | --- | --- | --- | --- | --- |
| Reckweg et al 2021 | 5-MEO-DMT (Inhalation) | 2.00 | 1 | NR | 0.37 (0.03-0.69) | NR | NR | NR | NR | NR | NR |
| Reckweg et al 2021 |  | 6.00 |  |  | 0.35 (0.09-0.64) |  |  |  |  |  |  |
| Reckweg et al 2021 |  | 12.00 |  |  | 0.20 (0.13-00.29) |  |  |  |  |  |  |
| Reckweg et al 2021 |  | 18.00 |  |  | 0.97 (0.15-2.42) |  |  |  |  |  |  |
| Rucker et al 2024 | 5-MEO-DMT (Intranasal) | 1.00 | 4 | 12 | 5.9 ±1.30 | {0.07}  (0.07-0.17) | 1.4 ±0.40 | 1.9 ±0.30 | 0.25 ±0.13 | 543 ±94.0 | NR |
| Rucker et al 2024 |  | 2.50 |  |  | 7.9 ±3.40 | {0.17}  (0.10-0.27) | 3.8 ±1.90 | 4.40 ±2.10 | 0.33 ±0.10 | 651 ±256 |  |
| Rucker et al 2024 |  | 4.00 |  |  | 8.7 ±4.70 | {0.13}  (0.05-0.17) | 4.5 ±2.20 | 5 ±2.20 | 0.38 ±0.13 | 953 ±511 |  |
| Rucker et al 2024 |  | 6.00 |  |  | 14.9 ±5.10 | {0.13}  (0.07-0.27) | 8 ±2.50 | 9.8 ±1.30 | 0.37 ±0.05 | 618 ±80.0 |  |
| Rucker et al 2024 |  | 8.00 |  |  | 22.1 ±5.50 | {0.17} (0.10-0.32) | 13.2 ±3.70 | 14 ±4.20 | 0.30 ±0.07 | 619 ±196 |  |
| Rucker et al 2024 |  | 10.0 |  |  | 32.3 ±15.0 | {0.10} (0.03-0.27) | 14.3 ±4.60 | 15.4 ±4.40 | 0.38 ±0.13 | 699 ±228 |  |
| Rucker et al 2024 |  | 12.0 |  |  | 28.9 ±7.20 | {0.25} (0.17-0.50) | 18.2 ±4.00 | 23.7 ±4.7 | 0.44 ±0.15 | 522 ±115 |  |

{Median}; (95%CI)

**Supplementary Material 9b –** Scatter plots with regression lines of pharmacokinetic parameters for Intranasal 5-MEO-DMT

Pharmacokinetic parameters (Cmax, Tmax, T½, AUC, clearance, volume distribution) are plotted against dose as circular scatter points. Lines represent simple linear regression fits weighted by trial arm sample size (n). In subplot a (Tmax & T½), Tmax is shown with blue circles and a navy regression line, while T½ is shown with orange circles and a red regression line. In subplots b–f, pharmacokinetic parameters are shown with light blue circles and orange regression lines No subplot for volume distribution is available for 5-MeO-DMT, as this was not reported in Rucker et al., 2024.

**Supplementary Material 10 -** Summary Table of Intravenous LSD Tartrate and Intravenous Psilocybin Pharmacokinetic Parameters

| **Study Reference** | **Compound** | **Dose (mg) \| Infusion rate** | **N Analysed** | **PK Session (Hours)** | **Cmax (ng/mL)** | **Tmax (Hours)** | **AUC 0-t (ng*h/mL)** | **AUC 0-inf (ng*h/mL)** | **T 1/2  (Hours)** | **Clearance (L/h)** | **Volume Distribution Vz/F (L)** |
| --- | --- | --- | --- | --- | --- | --- | --- | --- | --- | --- | --- |
| Arikci et al 2025 | LSD Tartrate (I.V) | 0.080\|0.009/min | 20 | 24 | 5.94 [4.95-7.12] | 0.16 [0.15-0.17] | 16.8 [14.60-19.30] | 17.2 [14.90-20.00] | 3.8 [3.60-4.10] | 4.7 [4.10-5.40] | 26 [23.0-29.0] |
| Hasler et al 1997 | Psilocybin (I.V) | 1.00 [30s bolus] | 6 | 2 | 12.9 ±5.60 | 0.03 ±0.02 | NR | 4.00 ±0.92 | 1.24 ±0.33 | 187.6ª ±43.1ª | 277☨ ±92.0☨ |

* Converted from pg*h/mL; ª Converted from (mL/min); ☨ VDss (L)
